# A proactive technique for reversal of Hartmann’s procedure: lifting the rectal stump to the abdominal wall

**DOI:** 10.1007/s10151-025-03128-0

**Published:** 2025-03-24

**Authors:** A. Fukada, T. Ogino, Y. Fujimoto, Y. Sekido, M. Takeda, T. Hata, A. Hamabe, N. Miyoshi, M. Uemura, T. Mizushima, H. Eguchi, Y. Doki

**Affiliations:** 1 Department of Gastroenterological Surgery, Graduate School of Medicine, Osaka University, 2-2 (E2) Yamadaoka, Suita, Osaka 5650871 Japan; 2 Department of Gastroenterological Surgery, Osaka Police Hospital, Osaka, Japan

**Keywords:** Hartmann’s procedure, Reversal of Hartmann’s procedure, Surgery, Diverticulitis, Laparoscopy

## Abstract

**Background:**

Reversing Hartmann’s procedure is complicated owing to dense adhesions resulting from inflammation in the pelvic region. These adhesions pose challenges in identifying the rectum and increase the risk of pelvic organ injuries.

**Methods:**

We propose a technique to lift and fix the rectal stump to the abdominal wall to diminish adhesions to the rectum and facilitate identification of the rectal stump.

**Results:**

The patient underwent Hartmann’s procedure for generalized peritonitis resulting from perforation of the sigmoid colon. The abdominal cavity was significantly contaminated with fecal ascites, and postoperative pelvic adhesions were anticipated. Therefore, the rectal stump was lifted. The outcomes demonstrated that despite the presence of dense adhesions in the abdominal cavity, the rectal segment was promptly identified during the reversal of Hartmann’s procedure. The procedure proceeded smoothly and was deemed satisfactory.

**Conclusions:**

The technique of lifting and fixing the rectal stump to the abdominal wall is useful in cases where dense pelvic adhesions are anticipated during the subsequent reversal of Hartmann’s procedure.

## Introduction

Hartmann’s procedure (HP) involves the resection of the diseased left-sided colon, accompanied by the creation of a proximal end colostomy and suture closure of the distal rectal stump. HP is typically reserved for emergency cases of left-sided colonic diseases, such as complicated diverticulitis, obstructing or perforated left-sided colonic tumors, and traumatic injuries associated with fecal contamination. In these high-risk emergency patients, HP is effective in circumventing the complexities associated with rectal anastomosis and avoiding postoperative anastomotic complications. Several studies have suggested that peritoneal lavage or primary anastomosis with a diverting ileostomy for perforated diverticulitis is preferable to HP in particular patients [1–3]. However, prioritizing sepsis control and devising surgical strategies to manage damage are paramount to ensuring patient survival. Additionally, maintaining intestinal continuity can be challenging in cases with compromised intestinal status; hence, HP is frequently chosen in emergency situations.

The reversal of colostomy after HP, known as the reversal of Hartmann’s procedure (RHP), poses a significant challenge. Severe inflammation after RHP can lead to intraabdominal adhesions and residual rectal atrophy. Consequently, tasks such as adhesiolysis, identification of the rectal stump, and anastomosis are technically demanding during RHP. Recently, laparoscopic surgery has become increasingly favored for nonmalignant surgeries [4]. Many studies have reported that laparoscopic RHP offers superior outcomes compared with laparotomy, including faster recovery and improved outcomes [5–7]. However, a notable challenge of laparoscopic RHP is the high rate of conversion to open surgery. The conversion rate is reported to be approximately 12%, with extensive adhesions being the most common cause, followed by factors related to the rectal stump [8]. Surgeons frequently encounter difficulties in identifying the rectal stump during RHP because it may retract into the lower pelvis and become obscured by fibrotic tissue. Challenges in identifying rectal stumps induced by rectal atrophy or severe pelvic adhesions can also impede the successful completion of RHP.

To address the challenges associated with RHP, we propose a novel technique for laparoscopic RHP, where the rectal stump is elevated to the anterior abdominal wall.

### Technical description

**Fig. 1:**
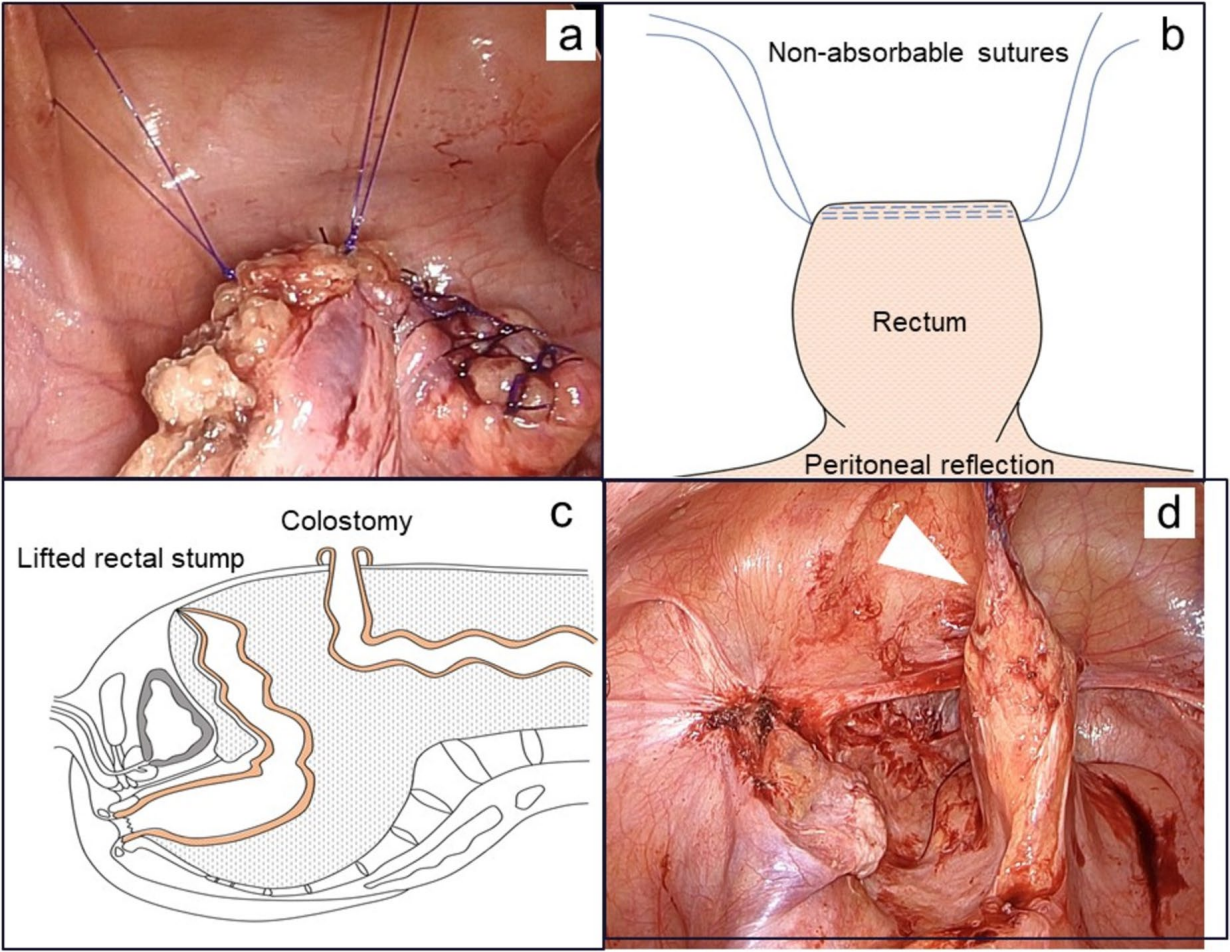
**a** Rectal stump was lifted to the abdominal wall using sutures in the HP. **b** Two points near the rectal stump were stitched with nonabsorbable sutures, which were left long enough for elevation. **c** Rectum fixed to the abdominal wall was confirmed to be lifted during RHP. **d** Triangular arrow indicates lifted rectum

This procedure aims to facilitate easy identification of the rectal stump during RHP by lifting it during the initial HP and fixing it to the abdominal wall. Initially, two sites of firm tissue near the rectal stump were selected, and nonabsorbable threads were stitched to each site (Fig. 1a, b). Subsequently, the sutured nonabsorbable threads were percutaneously retracted using Endoclose (Medtronic Inc., MN, USA) and fixed to the abdominal wall at the right and left positions just cephalad to the pubic bone (Fig. 1c). It is crucial to note the position at which the nonabsorbable threads are sewn to exclude vulnerable areas and prevent tissue tearing under tension during elevation. Preserving the rectal stump as much as possible is vital, as it serves as an important site for later anastomosis. Additionally, when pulling up a nonabsorbable thread, the rectum should be carefully handled to avoid exerting excessive tension on the tissue. Subsequent RHP is typically performed a few months after a favorable postoperative course.

**Fig. 2:**
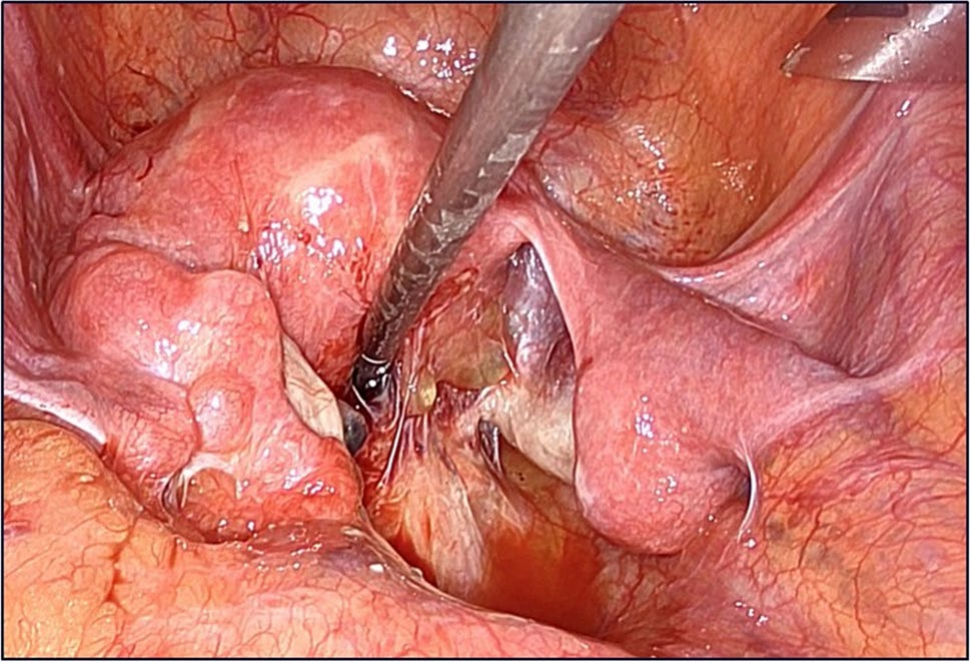
Intricate adhesions in the pelvic cavity make it difficult to identify the buried rectal segment. Adhesions are particularly severe in the Douglas fossa, and careful debridement is required to prevent injury to the uterus, ureters, and blood vessels

During the subsequent RHP, the rectal stump fixed to the abdominal wall in the previous operation was held in a lifted position, facilitating easy identification of the rectum (Fig. 1d). Additionally, unnecessary maneuvers for adhesion dissection are reduced, thereby minimizing the risk of organ damage. Without fixation, rectal stumps are covered by other pelvic organs and firmly adhere to the surrounding tissue, requiring extensive adhesion dissection (Fig. 2). This technique can be easily performed with minimal effort to relieve these burdens. After detaching the fixed threads and ensuring sufficient rectal mobility, laparoscopic rectal anastomosis was performed.

### Case presentation

**Fig. 3:**
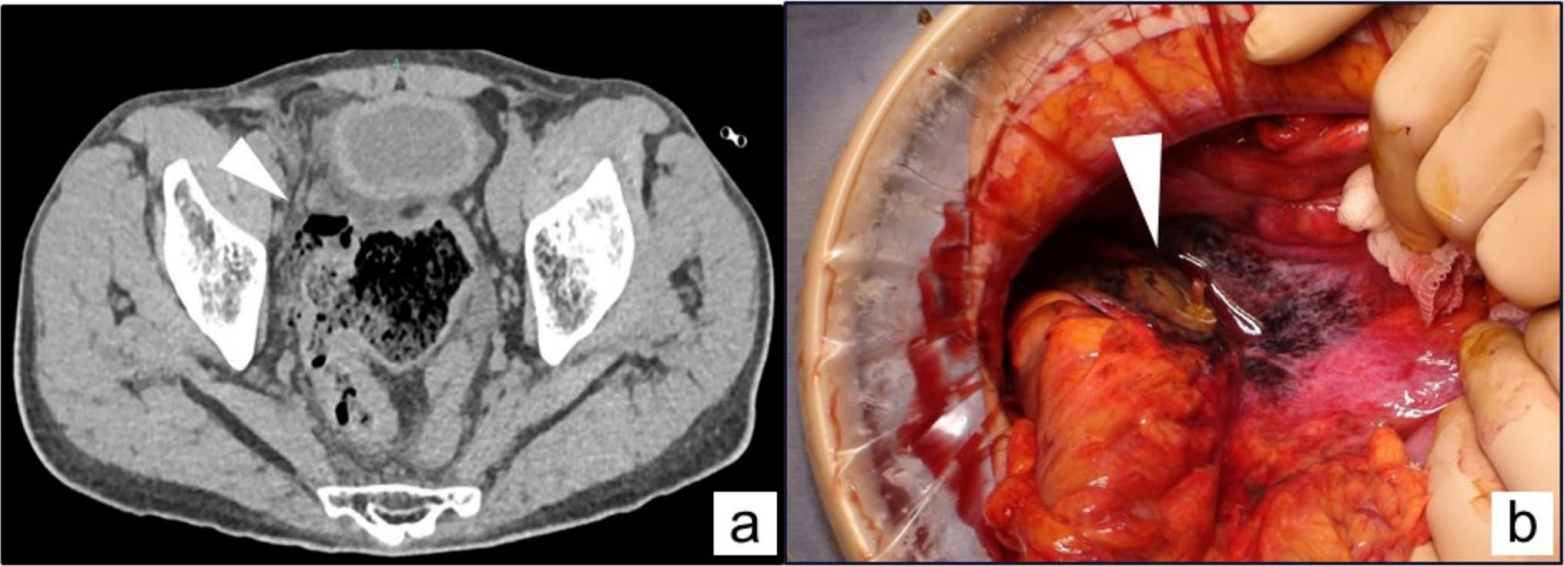
**a** Contrast-enhanced computed tomography showed free air around the sigmoid colon, as indicated by a triangular arrow. **b** Intraoperatively, the perforation of the sigmoid colon was identified, with the perforation indicated by the triangular arrow

**Fig. 4:**
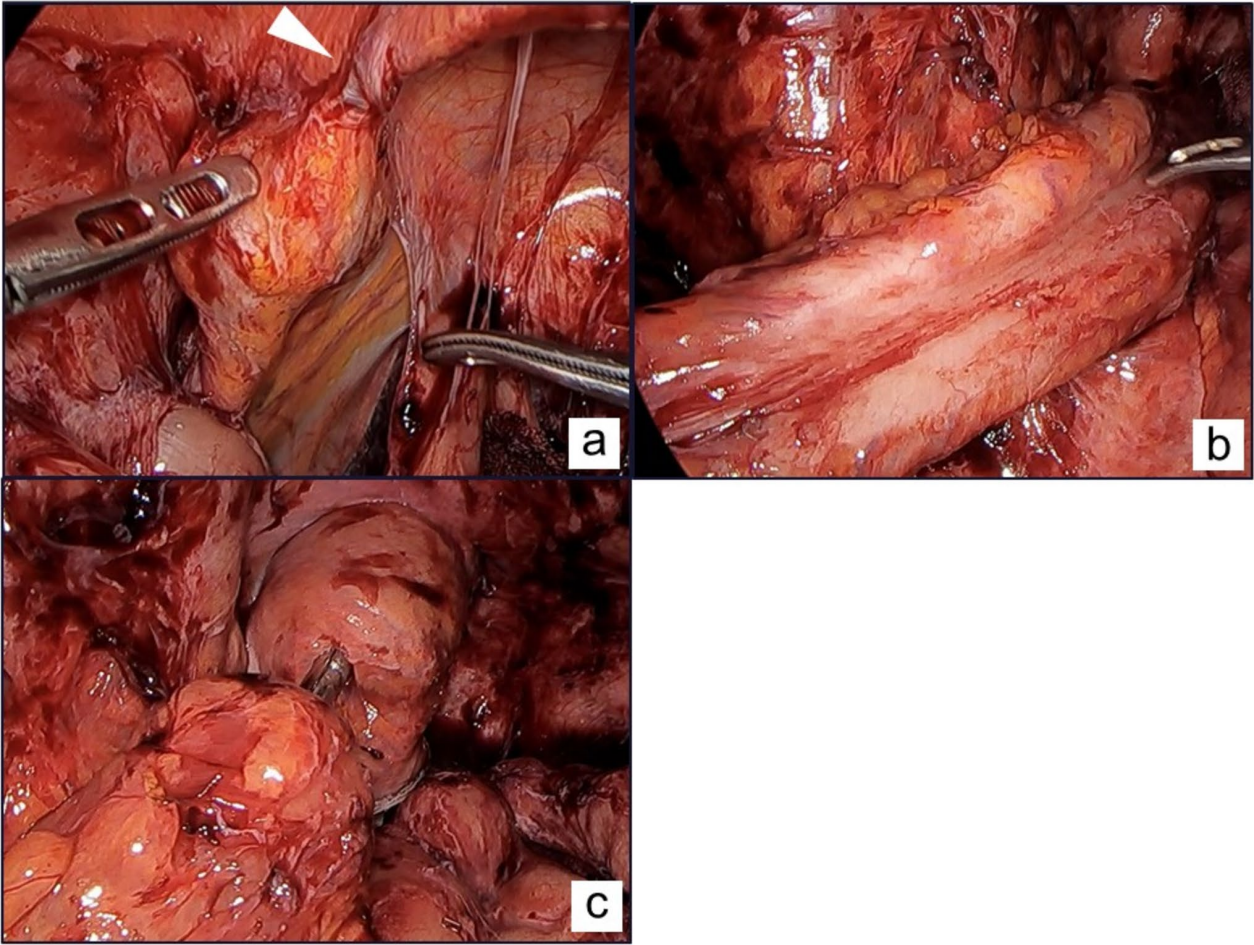
**a** The rectal stump, lifted to the abdominal wall, was easily detected after detaching the abdominal wall adhesions. A triangular arrow indicates the nonabsorbable sutures fixed to the abdominal wall. **b** Although the rectum had slight adhesion, it was easily mobilized. **c** Anastomosis was performed using the double stapling technique

A 63-year-old man presented to our hospital with lower abdominal pain and signs of peritoneal irritation. Contrast-enhanced computed tomography showed free air around the sigmoid colon and liver surface (Fig. 3a). In response to a diagnosis of diffuse peritonitis caused by perforated sigmoid diverticulitis, an emergency HP was performed (Fig. 3b). At the end of the surgery, the rectal stump was firmly lifted and fixed to the abdominal wall, and 4 months later, he underwent laparoscopic RHP. After the colostomy was taken down, the laparoscopic procedure was commenced. The patient developed generalized peritonitis postoperatively and exhibited extensive adhesions within the abdominal cavity. Upon dissection of the abdominal wall adhesions, the rectal stump, lifted to the anterior abdominal wall, was easily detected (Fig. 4a). The rectum was mobilized near the peritoneal reflection with adhesions in the pelvic cavity detached (Fig. 4b). The rectal stump was resected at the level of the promontorium, and colorectal intracorporeal anastomosis was performed using the double stapling technique (Fig. 4c). The postoperative course was uneventful, and the patient was discharged without complications.

## Discussion

HP is performed in patients with poor general conditions or at high risk of anastomotic leakage. Primary anastomosis is typically avoided in cases of severe inflammation of the pelvic cavity and edema of the residual rectal wall. RHP was subsequently performed after the patient’s condition stabilized and upon request.

RHP has been performed through laparotomy; however, recently, it has been increasingly performed laparoscopically. Laparoscopic RHP offers advantages over open surgery, including reduced postoperative pain, shorter hospitalization, and fewer postoperative complications [9]. However, this requires technical proficiency and can be complex. Identifying the rectal stump is essential during RHP. Most patients undergoing HP present with purulent or fecal peritonitis, which leads to significant adhesions in the pelvic cavity. These adhesions can obscure the rectal stump, which may become atrophic and retract deep into the pelvis. Separating the rectal stump from the surrounding pelvic viscera, such as the bladder, uterus, and vagina, can be challenging. Even when the rectal stump is marked with a nonabsorbable suture material during HP, it can be difficult to identify the rectum because of adhesions. In such cases, careful procedures are required to avoid serious complications, including pelvic organ injuries and bleeding. Presacral venous bleeding during rectal mobilization is uncommon but can be challenging to control and potentially life-threatening.

When laparoscopic dissection of adhesions is not feasible, open conversion is necessary. Previous reports have indicated an open conversion rate of 9–50% in laparoscopic RHP [7]. In most cases, the need for conversion is attributed to intraabdominal adhesions, difficult rectal identification, and rectal damage [8]. For safe surgery, it is necessary to overcome the problem of ensuring a rectal stump. Surgeons have introduced several innovations to solve the problems with RHP [10], such as fixing the rectal stump to the fascia of the anterior sacral surface [11], using an endoscope inserted through the anus to provide light for rectal stump identification [12], and retrograde injection of saline through a urethral balloon to delineate rectal boundaries [13].

Lifting the rectal stump to the abdominal wall offers several advantages. First, it facilitates the identification of the rectal stump, as it is fixed to the abdominal wall. The identified rectum is a marker when performing intraperitoneal adhesion dissection, which may reduce the risk of accidental organ injury. Second, it helps reduce adhesions around the rectal stump, thereby decreasing the risk of rectal injury and surgeon stress. Third, it may lessen rectal atrophy. In patients with rectal atrophy, it is necessary to mobilize the rectum deep in the pelvic region to facilitate anastomosis. The most important aspect of RHP is avoiding rectal injury and performing safe anastomoses. We have also applied the lifting technique to staged surgeries for ulcerative colitis (UC). Some patients with UC initially underwent subtotal colectomy as an emergency surgery for acute exacerbation, followed by residual rectum resection and ileal-pouch anal anastomosis as a secondary surgery. During the second-stage residual rectum resection, identifying and dissecting the remnant rectum can be challenging. In such cases, lifting the rectal stump at the first-stage subtotal colectomy facilitates easier identification of the rectal stump during the second-stage surgery. In one case involving a fragile residual rectum, rectal injury occurred due to tension from the lifting suture, necessitating additional resection of the rectal stump.

In cases where the residual rectal length is insufficient or the rectum cannot be lifted adequately, fixing it to the abdominal wall is challenging. Additionally, this technique may be unsuitable if the rectal tissues are fragile, as it may place tension on the rectum during lifting. In our experience, all subsequent surgeries following the lifting procedure were performed laparoscopically, with no conversions to open surgery; however, further studies are required to confirm its efficacy.

## Conclusions

We describe a new technique for laparoscopic RHP in which the rectal stump is elevated to the abdominal wall. This technique could be beneficial for reducing the complexity of RHP. However, further studies are required to confirm its efficacy.

## Data Availability

No datasets were generated or analyzed during the current study.
